# Australian aid projects: What works, where projects work and how Australia compares

**DOI:** 10.1002/app5.300

**Published:** 2020-05-11

**Authors:** Terence Wood, Sabit Otor, Matthew Dornan

**Affiliations:** ^1^ Development Policy Centre The Australian National University Canberra Australian Capital Territory Australia

**Keywords:** aid effectiveness, Australian aid, foreign aid, project effectiveness, the Pacific

## Abstract

In this article, we conduct the first‐ever systematic study of Australian aid project appraisals. Using a previously unstudied data set of appraisals, we study project and recipient country factors influencing Australian aid effectiveness. We find effectiveness varies more within recipient countries than between countries. We find larger projects are more likely to be successful. Humanitarian projects are more successful on average than development projects. We also find that Australian aid is less likely to succeed in the Pacific than elsewhere, a significant finding given Australia's increased focus on the region. Finally, we show that Australia does not appear to be an unusual donor: when we compare Australia with other donors in a global data set, we find similar variables are correlated with effectiveness for most donors, including Australia.

## 1 INTRODUCTION

At the project level, aid effectiveness varies considerably: some projects succeed whereas others do little good. Learning the ingredients of success is a crucial task for aid practitioners and researchers alike (Honig, 2018; Riddell, 2007). One means of shedding light on the ingredients of successful aid is studying project appraisals. Most aid agencies appraise the performance of their projects. When they do this, some agencies also produce numeric scores of project performance to accompany the qualitative components of appraisals. A growing body of international research makes use of the quantitative data emerging from these scores to study aid effectiveness. This quantitative work is no substitute for the detailed project‐specific learnings that can emerge from evaluations, yet comparisons across projects can answer important broader questions about aid effectiveness. Such analysis can provide insights into questions such as: which types of country projects are more likely to succeed in and what types of work are more likely to succeed (Feeny & Vuong, 2017). Quantitative analysis of project appraisal data can also allow for certain types of comparisons between aid agencies (Briggs, 2019).

Although the quantitative analysis of project performance is growing internationally, and although data amenable for analysis are produced for Australian government aid, Australian aid effectiveness has never been studied in this way. In this article, we present the first‐ever analysis of a new data set of 456 Australian Government Aid Program projects. Among other findings, we show that Australian aid appears to be at its least effective in the Pacific. This is concerning given the importance of aid to the region—11 of the world's 20 most aid‐dependent countries are in the Pacific (Organisation for Economic Co‐operation and Development [OECD], 2019) 1Aid dependency was calculated as aid/gross national income, a standard measure, and was taken from 2017, the most recent year with full global data at the time of writing.—and the fact that Australia is by far the largest donor to the Pacific (Dayant & Pryke, 2019). From an Australian perspective, underperformance in the Pacific is concerning given Australia devotes more aid to the Pacific than any other region. 2In 2017–18, the most recent year for which data are available, Australia devoted 27%, which is the plurality, of its total aid spend to the Pacific. Data from: https://dfat.gov.au/about-us/publications/Documents/2017-18-std-time-series-table-4-partner-country.xlsx. Australia's recent move into aid loans to the Pacific adds further to this concern (Wood & Otor, 2019).

Yet, while recipient country traits are associated with whether projects succeed or not, we also find that most of the variation in Australian aid project effectiveness occurs within countries rather than between them. Project quality matters more for successful aid than recipient country context. The Pacific poses a challenge for Australian aid effectiveness. Yet our findings suggest it is a surmountable challenge if projects are appropriately designed.

In our own study of the project‐level traits that affect Australian aid effectiveness, we are constrained by limited available data on project specifics. Working with the data available to us, we find that larger projects are more likely to succeed, as is aid given as part of humanitarian emergency responses.

Finally, when we compare Australia with other donors that have produced similar data, we find Australia does not differ radically from other donors in terms of how country and project traits are associated with aid effectiveness.

The rest of this article is structured as follows. After summarising existing research, and detailing research methods, we focus on the question of whether the effectiveness of individual Australian aid projects changes over time. We then look at correlates of project success—what types of projects were more likely to be appraised as successful and where projects were more likely to be appraised as successful. We subsequently look at the effectiveness of aid projects in the Pacific. Finally, we situate Australian Aid Program data within a new, growing data set of project appraisals from other aid donors, comparing the correlates of success across donors.

## 2 WHAT CAN BE LEARNT ABOUT AID EFFECTIVENESS FROM PROJECT DATA?

For a long time, the World Bank was the only donor to make aid project appraisal data available in a form that was easily collatable and amenable to quantitative analysis. The World Bank was joined in 2017 by the Asian Development Bank (ADB), which provided a one‐off release of similar data.

As a result of the two banks' willingness to release data, early work involving aid project analysis focused almost exclusively on World Bank projects, with more recent analysis also focusing on ADB projects. Only in the past two years, courtesy of a remarkable data gathering effort from Professor Dan Honig, have other donors been analysed (Honig, 2018).

Key findings from earlier work were that, although recipient country features have an impact on aid project effectiveness, project success often varies as much within countries as between them, suggesting that project attributes play an important role in whether projects succeed or not. Of those country‐level factors found to influence project success, economic growth was often found to be positively associated with success. Although the relationship is somewhat more ambiguous, levels of recipient gross domestic product (GDP) have also been found at times to be associated with success (Bulman, Kolkma, & Kraay, 2017; Denizer, Kaufmann, & Kraay, 2013; Feeny & Vuong, 2017; Kilby, 2000). Generally, when it has been studied, better governance has been found to be positively associated with project success. However, the relationship between democracy and civil liberties, and success, appears more mixed. Some studies have found a positive relationship others have found no relationship or even a negative relationship (Bulman et al., 2017; Denizer et al., 2013; Feeny & Vuong, 2017; Isham, Kaufmann, & Pritchett, 1997). Negative findings for democratic freedoms emerge more often in work focused on the Asia‐Pacific region.

Many studies have also examined project‐level variables such as size and sector (Bulman et al., 2017; Denizer et al., 2013; Feeny & Vuong, 2017). A fair conclusion would be that where these traits have been studied, findings have been mixed—no clear consensus has emerged, for example, that certain sectors are more likely to succeed. At least two studies have found projects that were longer in duration were usually less favourably appraised although the findings are not always present, and challenges exist in isolating cause from effect in this particular relationship (Denizer et al., 2013; Feeny & Vuong, 2017).

Interesting variations on existing studies can be found in Kilby (2000) who found more staff supervision of projects was associated with better outcomes in World Bank work, and Honig (2018) who found that projects were more likely to succeed in difficult environments when in‐country practitioners were less constrained by directives from central management, and freer to adapt to context. One recent study (Briggs, 2019) has shown that, although most earlier work has only been based on two donors (the World Bank and ADB), at least some important findings appear to generalise across the broader set of donors included in Honig's (2018) data set.

## 3 DATA

The data used both in existing research and this article come from donor's own project appraisals. These appraisals come from a range of different sources, from external reviews to internal assessments. In the Australian case, as with most other donors, appraisals are normally conducted by aid program staff. This brings an obvious concern: it may be in donors' interests to provide unduly positive appraisals. Usually, in the Australian case, project duration and staff rotation mean that appraisers are not appraising the projects they themselves set up or advocated for. There are also clear criteria in appraisal guidelines that staff are meant to follow when providing scores. Scores are meant to be commensurate with detailed qualitative descriptions of project performance that are included in appraisal documents. However, incentives may well still exist for staff to provide overly kind scores. Some reassurance in this area can be found in Denizer et al.’s 2013 analysis of World Bank data, which shows that, on average, projects appraised through internal assessments (the same process that produced the Australian data) did not tend to receive higher scores than those appraised through rigorous independent reviews. However, there remains a clear possibility that absolute scores of project effectiveness will be too high.

Fortunately, even with this risk, appraisal data retain some utility. As long as it can be safely assumed that the extent to which projects are overly positively appraised is constant across projects, comparisons between projects will still allow for the correct identification of factors that contribute to project success. There are two obvious cases where inflation in project assessments will not be constant between projects. One is where projects come from different donors (different norms or standards may lead to differing degrees of inflation). Another is where projects come from different time periods: it is possible that donors may be kinder in their appraisals in certain times, when under increased political scrutiny for example. Fortunately, both of these risks can be controlled for in regression analysis by adding donor and completion year fixed effects to regression models.

A further risk is that aid in some recipients may be evaluated more positively because of the geopolitical significance of those countries. There is evidence geopolitical considerations affect World Bank aid project appraisals (Kilby & Michaelowa, 2016). In Australia's case, as we discuss in Supporting Information Appendix 3, appraisals were unexpectedly positive in Papua New Guinea. (All Supporting Information Appendices can be accessed at: http://doi.org/10.6084/m9.figshare.11678118.) Of all the countries in the Pacific, the mean appraisal is second highest in Papua New Guinea, which will likely come as a surprise to anyone who has worked in that country's challenging context. Plausibly, the high ratings could reflect Australia's long experience as a donor in Papua New Guinea. However, findings could also reflect the sensitive nature of the political relationship between Papua New Guinea and Australia. Somewhat reassuringly, Nauru, a similarly strategic country, has the worst average appraisal of all Pacific countries, suggesting that intergovernmental relations do not always drive Australian project appraisals. As a robustness test we re‐ran the Australia‐focused regressions from this article with Papua New Guinea excluded. These are included in Supporting Information Appendix 3. As can be seen, results changed little when Papua New Guinea was excluded. Even with these precautions, it is plausible that some other bias, which we cannot control for, possibly associated with sectoral differences, for example, still exists. There is no *prima facie* reason to believe such a bias will exist. However, it is a possibility, and this is a limitation of our work, alongside most other work drawing on project appraisals. We believe the utility of analysing appraisal data is sufficient to justify this risk.

The Australian data that we use come from three slightly different sources: Aid Quality Checks (AQCs), Final Aid Quality Checks (FAQCs), and Humanitarian Aid Quality Checks (HAQCs). AQCs are conducted throughout the lifetime of a project; FAQCs are conducted at project completion, and HAQCs are similar to FAQCs but used only for humanitarian emergency projects. The data are all in the public domain and were provided to us in collated form by the Australian Government Aid Program. All three document types are similar and appraise a range of project attributes on a 1 to 6 scale. All of the projects in the Australian data set we use were appraised between 2013 and 2019. Almost all of the projects started after 2005. Except in the first analytical section of this article, which tracks change over time, we have based our analysis on projects' most recent quality check scores only. Conforming with practice used in similar international studies, no project is included more than once in our analysis. For non‐humanitarian projects that have finished, the most recent score is usually an FAQC score.

Although AQC, FAQC and HAQC reports appraise projects on a range of attributes, in the main body of this document, we focus only on project effectiveness. We do so for two reasons: effectiveness is a better match with the attributes studied in most other similar papers; and focusing on only one attribute allows for more readily interpreted analysis. In Supporting Information Appendix 2, we study the extent to which there is a correlation between different project attributes in appraisals (we find clear correlations). Also, because country traits (such as governance and GDP growth) cannot be obtained for regions, all of the analysis in the main body of this work is restricted to country‐level aid projects; regionwide aid projects are excluded.

Compilation of the data used in this project involved first taking Australian project data 3Australian data can be found in the datafile at: http://doi.org/10.6084/m9.figshare.11678118. and integrating it with World Bank 4World Bank data can be accessed at: https://finances.worldbank.org/Other/IEG-World-Bank-Project-Performance-Ratings/rq9d-pctf. and ADB data. 5ADB data can be accessed at: https://www.adb.org/sites/default/files/evaluation-document/214201/files/2017-aer-rating-database.xlsx. These data were then appended to the international donor data set created by Professor Honig. 6Honig's dataset can be accessed at: https://danhonig.info/. Honig's data set contained data for the following donors: the UK's Department for International Development (DFID); Deutsche Gesellschaft für Internationale Zusammenarbeit (GiZ), the German government's development agency; KfW, the German government's development bank; the International Fund for Agricultural Development (a United Nations [UN] institution; hereafter IFAD); Japan International Cooperation Agency (the Japanese government aid program; hereafter JICA); and The Global Fund to Fight AIDS, Tuberculosis and Malaria (hereafter GFATM or Global Fund). Honig's data set contained project assessment scores standardised to a six‐point scale (the same scale used by the Australian Government Aid Program). The Honig data set also contained project start and end dates, project size and information on project sectors. (Once again, we had similar information for Australian projects.) Although Honig's data set also contained data for the World Bank and ADB, we chose to use our own data as they contained more recent projects. We standardised data such as projects' sectors across all these different sources.

Next, we took the combined project data set and imported World Bank World Development Indicator data on recipient country economic and demographic indicators, World Bank Country Policy and Institutional Assessment (CPIA) data, World Bank government effectiveness data, and Freedom House data on political and civil freedoms. Then, for each of the recipient country variables from these data sets, we calculated averages for the lifetimes of each individual project. (For example, if a project ran in Papua New Guinea from 1980 to 1985, for the inflation variable, we calculated the mean inflation in Papua New Guinea from 1980 to 1985.)

Although we only needed Australian and recipient country data to conduct the bulk of the analysis reported in this article, the international data set afforded the ability to compare Australia with other donors. The resulting data set contained at least some information for nine donors and 182 recipient countries. (Full details of donors and projects can be found in Supporting Information Appendix 1.) Some donors are much better covered than others in the data set. The World Bank is by far the best; Australia has fewer projects but is still relatively well represented.

Although some of the Australian projects in the data set started in the 1980s, they are very few in number. Almost all of the evaluated Australian projects started after 2005. All of the quality control reports that contributed to the data we were able to use were conducted after 2013 and the integration of AusAID into the Department of Foreign Affairs and Trade.

## 4 AUSTRALIAN AID

### 4.1 The distribution of appraisal scores

**FIGURE 1:**
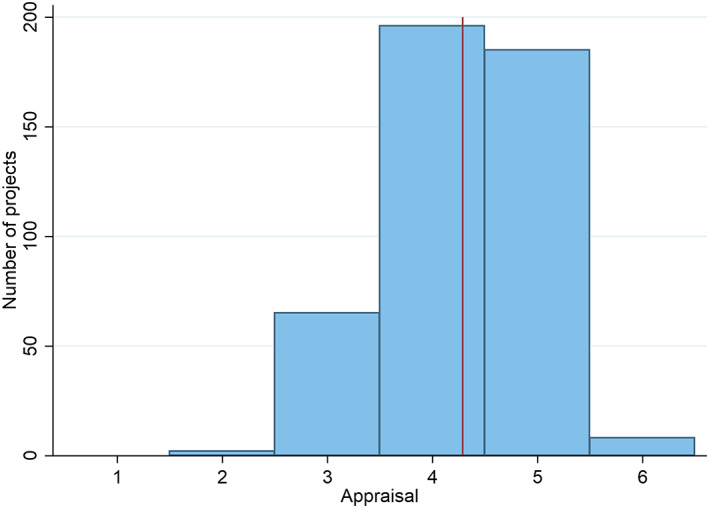
Australian aid project effectiveness appraisals

Figure 1 is a histogram showing how Australian aid project effectiveness ratings are distributed from 1 to 6. The vertical line is the mean.

There is a clear aversion to appraising projects with a rating of 1 or 2. There is a similar aversion to rating projects a 6. For reasons outlined above, we cannot conclude from these scores that Australian aid projects never fail miserably. It is just as likely the case that people completing appraisal forms are reluctant to admit to failure. It is also worth noting that a score of 3 is still considered an unsatisfactory outcome in the Australian Government Aid Program's reporting guidance and is meant to trigger remedial action. Moreover, while the data cluster around the mean there is still some range; it is not the case that all projects receive the same score. There is enough variation to conduct analysis, although there would be more leverage still if more projects were graded with 1s, 2s and 6s. Importantly, as is shown in Supporting Information Appendix 1, Australia is far from unique in its distribution of effectiveness scores. Most other donors are similarly reluctant to score projects very well or very poorly.

### 4.2 Do projects' appraisals change much over time?

One question of interest is how often project effectiveness changes substantially over a project's lifetime? Do projects that start poorly ever recover to deliver great benefits? Do projects that start well ever subsequently go astray. The question is of clear policy relevance. If project quality rarely changes over time, there is a case for discontinuing poor‐performing projects early in their lifespans. The following analysis compares Australian projects' earliest scores in the data set with their most recent scores. The comparison is made regardless of the type of quality checks that were earliest and most recent (that is, both AQC and FAQC appraisals are used as needed). Because humanitarian interventions are likely to be something of a special case in terms of change over time (they are shorter and more discrete) HAQC appraisals were excluded from the analysis.

**FIGURE 2:**
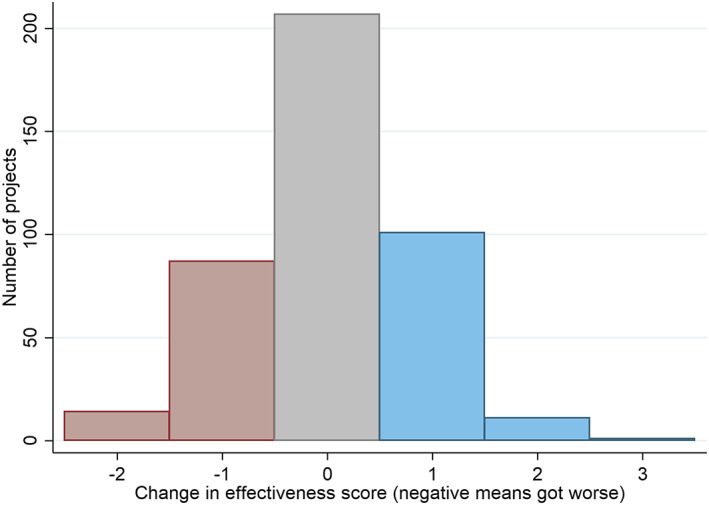
Change in aid project effectiveness appraisals

The histogram in Figure 2 shows change between the first and most recent project appraisals. The x‐axis shows the change in project effectiveness scores. The y‐axis shows the number of projects that changed by that amount. Red indicates deterioration, grey no change and blue improvement.

As the histogram shows, the appraisal of the modal project did not change. However, nearly 50% of projects did change, with positive change slightly more common than negative change. The fortunes of many projects do not appear to be entirely static across their lifespans. Change is, however, almost never larger than two points on a 1 to 6 scale. This would seem to suggest that transformation of fortunes is very rare, although given the way projects cluster around scores of 4 and 5 in Figure 1, it may well be the case that real‐world change is much larger than changes in appraisal scores suggest.

Because the time between the first appraisal in the data set and the most recent is not the same for all projects, we tested formally to see whether there was a relationship between the time elapsed between the first appraisal and the most recent, and the magnitude of change. For the sake of succinctness, we have excluded these results. However, we found a clear relationship: as time between appraisals became greater the average change in appraisals also increased. Given time, project quality shifts more.

For policymakers, the findings in this section suggest poor‐performing projects often can be redeemed with sufficient effort. And success at times can turn to failure if sufficient attention is not paid to the causes of project success.

### 4.3 Good projects or good recipients

The rest of our analysis of Australian aid data draws solely on projects' most recent appraisals. This approach is in line with that found in most other studies and makes sense given that many project attributes do not vary over the project cycle.

One clear finding from studies of World Bank and ADB data is that there is more variation in project outcomes within countries than between countries (Briggs, 2019; Bulman et al., 2017; Denizer et al., 2013). We tested whether this was true or not on Australian data using a standard approach: assessing the *R*‐squared values of regressions run with country fixed effects added (Briggs, 2019; Denizer et al., 2013). Doing this effectively identifies all of the difference in project outcomes that can be attributed to differences between countries. Because countries change over time (they may, for example, become better or worse governed), we also re‐ran the model with project start and end year fixed effects included. 7The term ‘end year’ here refers either to the year the project ended or the year the most recent evaluation was conducted if the project is ongoing. Findings can be seen in Table 1. The *R*‐squared values in the two regressions are .07 and .13, suggesting that between 7% and 13% of the total variation in Australian aid project outcomes is a result of country‐level factors as opposed to attributes associated with projects. This finding is in line with other studies. Like other donors, Australian aid success appears to be more dependent on project characteristics than on country‐level factors.

**TABLE 1:** Variation in project outcomes stemming from differences between countries

	Country fixed effects	Country and year fixed effects
Country	Yes	Yes
Start FE	No	Yes
Completion FE	No	Yes
Observations	456	456
*R*‐squared	.07	.13

### 4.4 The correlates of Australian project effectiveness

Table 2 shows the results of multiple regressions that examine the relationship between various project and country‐level variables and appraised project success. The table includes models that focus solely on project variables, models that focus solely on recipient country traits and models that include both. One recognised issue in other work on other donors is the possibility that standards of project appraisal change over time (Denizer et al., 2013). For this reason, we include models that contain end year fixed effects as a means of accounting for changing appraisal standards. 8Because they provide readily interpretable results, ordinary least squares (OLS) regressions were used. However, as shown in Supporting Information Appendix 4, if ordered logistic regression models are used instead, substantive findings are similar.


**TABLE 2:** Correlates of project success

	Project	Project with FE	Country	Country with FE	Full	Full with FE
Project size (natural log)	.07^***^ (.03)	.07^**^ (.03)			.07^**^ (.03)	.06^**^ (.03)
Duration of project (days)	−.00 (.00)	−.00 (.00)			−.00 (.00)	−.00 (.00)
Sector (humanitarian omitted)						
Economic	−.24^**^ (.12)	−.23^*^ (.12)			−.24^**^ (.12)	−.22^*^ (.12)
Education	−.15 (.11)	−.17 (.11)			−.14 (.12)	−.15 (.12)
Governance	−.24^**^ (.11)	−.24^**^ (.12)			−.23^*^ (.12)	−.23^*^ (.12)
Health	−.22 (.13)	−.23^*^ (.14)			−.20 (.14)	−.20 (.14)
Other	−.25 (.18)	−.31^*^ (.18)			−.22 (.18)	−.28 (.18)
Freedom House liberties			−.02 (.02)	−.02 (.02)	−.01 (.02)	−.01 (.02)
World Bank govt effectiveness			.01 (.11)	.08 (.10)	.01 (.11)	.08 (.11)
Real GDP/capita growth			−.00 (.01)	−.00 (.02)	−.01 (.02)	−.01 (.02)
GDP per capita (1,000s)			.03^**^ (.01)	.03^**^ (.01)	.03^**^ (.02)	.03^*^ (.02)
Comp year fixed effects	No	Yes	No	Yes	No	Yes
Observations	456	456	450	450	450	450

*Note:* Huber–White robust standard errors in parentheses

* *p* < .10

** *p* < .05

*** *p* < .01

There is a clear relationship in all of the models between project size and appraisals—larger projects tend to be evaluated as more effective. This is a useful finding and provides evidence that Australian aid effectiveness will be increased if the Aid Program does not allow its work to become fragmented across too many small aid projects. One potential confounding factor exists for this finding, however. This is that size may be endogenous to project performance. Projects may become larger if they perform well in their early stages. To test for this, we examined all those projects for which we had multiple years of budget data, and which had their first appraisals during the period covered by our data set. We then tested to see if appraisal scores were in any way correlated to subsequent changes in budget sizes. Using a range of different approaches (full results available from the authors on request) we found no relationship between appraisals and subsequent changes in total project budgets. As best we can tell, the relationship between project size and project performance is being driven by larger projects performing better.

Compared with humanitarian projects, projects focused on economic development and governance tend to perform worse. 9When interpreting the sectoral results, it should be noted that Australian Aid Program sectors did not map as well as we would have liked to the OECD‐derived schema we used in our analysis. Also, we noted some projects were incorrectly coded to the wrong sectors in the Australian source data. These issues have probably not affected the results unduly. However, obtaining better sectoral data would represent an improvement. (In this section, we deem all relationships where the *p* value is consistently less than .1 across models as having sufficient statistical significance to warrant noting.) Australia's aid for humanitarian emergencies appears to be more successful on average than its longer‐term development projects.

Because there is value in knowing the sectors in which development projects were more likely to succeed, in work not shown here, we re‐ran the regressions with a different base category used as the reference for sectoral comparisons. When we did this, there were no statistically significant differences in performance across non‐humanitarian sectors. We found no clear evidence, for example, that governance projects were, on average, less successful than projects focused on economic development. The only difference worth mentioning can be observed in the size of the sectoral coefficients found in Table 2: this is that education projects tended to be appraised somewhat better on average. This is suggestive; however, the differences were not statistically significant. Possibly, education projects perform better, but at this point, evidence of this fact is only weak.

In line with the findings of the previous section, it was hard to find country traits that were correlated with success. Projects tend to be more successful in wealthier recipient countries, which is useful to know, although the finding presents the Australian Aid Program with a dilemma, given it should be targeting aid to poorer countries where the need is greater. No other country variables were correlated with project outcomes in a manner that was statistically significant. Statistical significance aside, it is worth noting the regression coefficients on two of the country variables have the expected signs based on other relevant work on other donors. If there is a relationship between government effectiveness and project success, it is quite possibly a positive one. If there is a relationship between civil and political liberties and project success, it may be a negative one.

Although these findings are useful, it is worth contrasting the comparatively limited project data we had available to us with the much richer data sets that have powered some studies (see, for example, Denizer et al., 2013; Honig, 2018). In future work, much may be added if more project‐level variables can be included in analysis.

### 4.5 Australian aid and the Pacific

**FIGURE 3:**
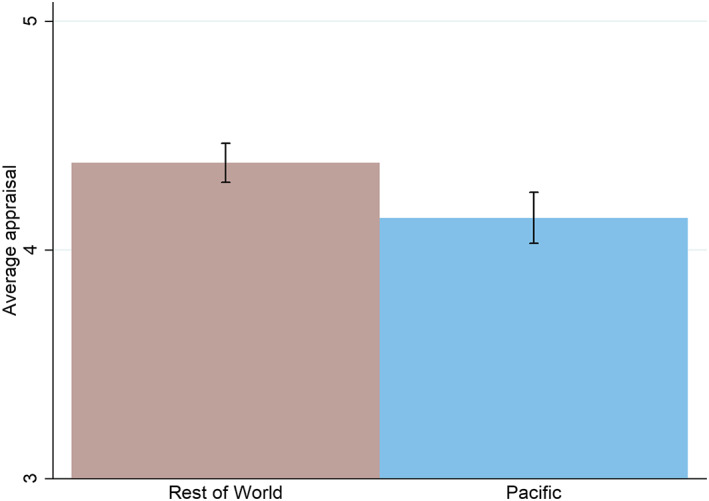
Project outcomes in the Pacific and elsewhere

Existing work, based on ADB and World Bank data, has shown that aid projects are less likely to succeed in the Pacific than in the rest of the developing world (Feeny & Vuong, 2017; Wood & Otor, 2019). Figure 3 displays similar analysis for Australian aid. The average appraisal of a project in the Pacific is worse than elsewhere. The difference is statistically significant. In an absolute sense the magnitude of the difference is not massive: only about one‐quarter of a point on a six‐point scale. However, it should be remembered that, as shown earlier, project appraisals tend to cluster together. The real difference may be understated by differences in appraisals. The difference also continues to exist (it is both statistically significant and of a very similar size) when all the controls used in the full regression model with fixed effects in Table 2 are accounted for. There is a stubborn gap in Australian aid project effectiveness between the Pacific and the rest of the world.

## 5 AUSTRALIAN AID COMPARED

Figure A1 in Supporting Information Appendix 1 shows histograms of project appraisal scores for different donors in our data set. 10Some donors' bars are situated between appraisal scores and there are gaps in the bars (for example, the ADB). These are donors whose own assessments of projects were not originally on 1 to 6 scales, and whose scores had to be rescaled to allow international comparisons. Rescaling was conducted in line with the advice in Honig's dataset. Each histogram also includes a red line showing the donor's mean score. There is considerable variation in donors' project appraisal scores, and some donors have higher means than others. This cannot, however, be taken to be indicative of varying performance between donors. It may be the case that different donors are more or less forgiving on their work in the subjective task of appraising it. Setting aside this concern, Australia would not seem to be an outlier. Its distribution of appraisal scores differs from the other donors, but not wildly so.

### 5.1 The Pacific effect: Australia and other donors

In his 2019 paper, Briggs undertakes innovative analysis comparing donors to see whether the same project and country traits have similar effects on project appraisals from different donors. In the following work we borrow from his approach to see how typical Australia is as an aid donor in terms of the correlates of project success.

**FIGURE 4:**
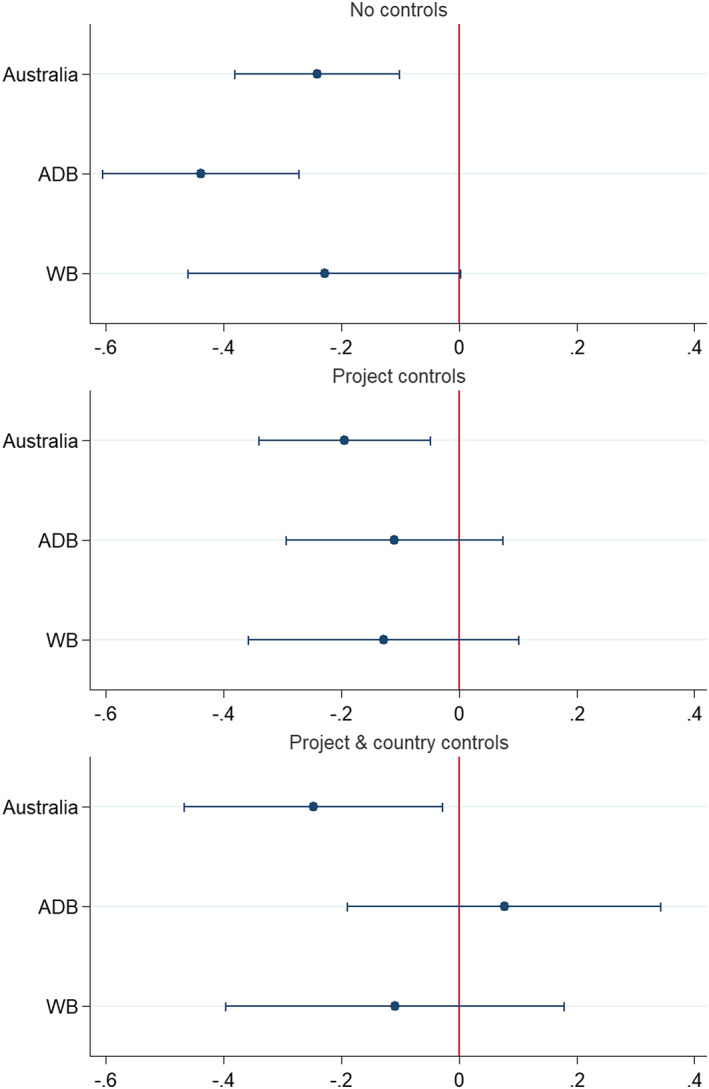
The Pacific effect by donor

Figure 4 answers the question of whether other donors struggle with worse project outcomes in the Pacific. The analysis is restricted to those donors with more than 100 Pacific projects. Only three of the donors in the data set fall into this category: Australia (172), the ADB (158) and the World Bank (111). Other donors, such as JICA, with only 15 Pacific projects in the data set, have too few projects to provide accurate estimates. Each donor is plotted on the chart. In each panel of the chart the regression coefficient for a Pacific dummy variable is plotted for each donor, along with 95% confidence intervals. The first panel of the chart simply compares the average project score in the Pacific with the average project score elsewhere for each donor. The second panel shows estimates from a regression with project‐level controls (thereby accounting as best possible for the possibility projects might be different in the Pacific—smaller or in different sectors for example—and that this might be behind different performance in the Pacific). The third panel contains country‐level controls as well. In doing this, we test whether projects in the Pacific have different outcomes even when specific project and country attributes are accounted for.

The first panel shows that all three donors experience worse project outcomes in the Pacific. In the second panel, outcomes for Australian projects stay statistically significant. Projects for the World Bank and ADB cease to be statistically significantly different from zero, although the point estimates are still negative—if anything—even when taking account of different project traits. Like Australia, the ADB and World Bank have less successful projects in the Pacific, although we cannot be as certain of this finding for the ADB and World Bank. In the third model, the same country controls used in Table 2 were added to the regression models. The effect of the Pacific for Australia is still negative and statistically significant. The effect for the World Bank is negative, but not statistically significant, and the estimate for the ADB is actually positive, although once again the estimate is not statistically significant.

Australia is not alone in experiencing worse project outcomes in the Pacific, although the Pacific effect on the World Bank and ADB is less readily apparent when controlling for potential differences in the nature of aid work in the Pacific and when controlling for the development traits of Pacific Island countries.

One final point needs to be noted regarding the findings in Figure 4. Although Australia's Pacific effect remains statistically significant when the same effect ceases to be statistically significant for the other two donors, this could simply stem from smaller samples for the other donors (they do not do as much work in the Pacific). Also, it needs to be emphasised that except for the difference between Australia and the ADB in the first and third models, the difference between donors is never itself statistically significant. The evidence regarding the Pacific effect being more severe for Australia is more suggestive than definitive. 11In the case of the ADB, even though differences are statistically significant, in one model the ADB performs worse, while in the other it performs better—its variance from Australia is not consistent.


### 5.2 The impact of GDP, project size, governance and freedom

**FIGURE 5:**
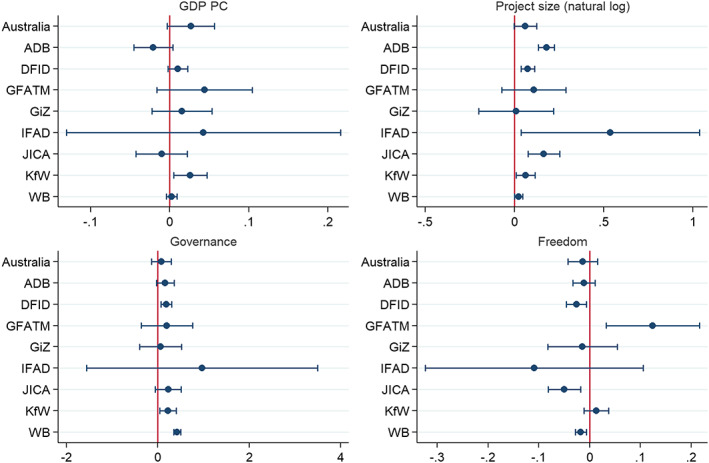
Relationships for GDP, project size, governance and freedom across donors

In Figure 5 the relationships between key variables from the literature and aid project success are compared across donors. This is done in a similar manner to Figure 4. Coefficients and 95% confidence intervals are shown for the relationship between the variable in question and project success for each donor. In all cases, the relationships are shown controlling for the other variables used in the full regression model above.

In the regression model above that looked at the country‐level correlates of Australian aid project success, GDP per capita proved to be one of the few country variables positively associated with project success in a manner that was statistically significant (*p* < .1). The first panel of Figure 5 shows the relationship between GDP and success for Australian as well as other donors.

Australia is not alone having more success in wealthier countries. As Figure 5 shows, most donors have more success, on average, when working in wealthier recipient countries. The only donors with negative coefficients for GDP are JICA and the ADB, and these relationships are not clearly statistically significant. The only clear—statistically significant—differences between Australia and other donors are between Australia and the ADB, and to a lesser extent JICA. Overall, Australia appears to be a fairly typical donor in finding projects easier to deliver successfully in wealthier countries.

The second panel of Figure 5 compares the relationship between project size and project success across donors. Once again Australia appears in line with most other donors. The relationship between project size and success is positive on average for all donors, albeit not statistically significant for some. The positive relationship that can be seen for Australia is similar in magnitude to that of all other donors except the ADB, JICA and IFAD, which display appreciably larger positive relationships.

The third panel of Figure 5 shows the relationship between recipient country government effectiveness (as per the World Bank governance indicators) and project success. Positive relationships exist for all donors, although they are not statistically significant for several, including Australia. The only donor whose coefficient is statistically significantly different from Australia is the World Bank, which appears to experience a particularly clear relationship between governance and project success. Notwithstanding this one difference, Australia appears to be a fairly typical donor with respect to recipient government effectiveness.

The fourth panel of Figure 5 shows the relationship between civil and political liberties and project success. There is an interesting degree of variation in the findings associated with liberties. In Australia's case, as with five other donors (the ADB, DFID, IFAD, JICA and the World Bank) the relationship is, if anything, likely to be negative. That is, projects perform better in less free countries, although for Australia, IFAD and the ADB the relationship is not statistically significant. Not all donors find project success negatively associated with freedom, however. For the Global Fund and KfW, coefficients are positive, albeit only statistically significant for the Global Fund. When it comes to the relationship between political and civil liberties and project success, Australia is not unique; however, there is diversity among donors, and some donors at least appear to have quite different experiences from Australia.

## 6 CONCLUSION

Quantitative analysis of aid project appraisals affords insights into Australian aid. When compared internationally, Australia does not appear to be an outlier—a country clearly different from others in terms of the project and country factors that contribute to the success of its aid.

Focusing solely on Australia, there are some useful learnings. Most important of these is that Australian aid projects are less successful in the Pacific. Although Australia is not necessarily alone in this, the ramifications of this finding are important given Australia's increased focus on the region, as well as the increasing Pacific focus of some other donors. For researchers using aid effectiveness data, there are two urgent tasks: learning what it is about the Pacific that leads aid to be less effective there and also ascertaining whether different types of aid work are more or less likely to succeed in the Pacific. For the Australian Government Aid Program, there are clear takeaways too. Because aid is less likely to succeed in the Pacific, the Australian Government should take extra care if, as it plans, it starts giving loans in the region. Aid projects funded through loans need to succeed if the loans are to be easily repaid. Learning how to make such loan‐funded projects succeed will be crucial. Also, there is a strong case for the Australian Government Aid Program taking a heavily learning‐oriented approach to its work in the Pacific. External researchers may well be able to learn more about the impediments to successful aid in the region, but the Aid Program itself can learn a lot by investing as much as possible in robust evaluations. The fact aid is less successful in the Pacific is not a counsel of despair: as we demonstrated in this article, project attributes appear to be more important to aid success than country and regional traits. If the Aid Program can better calibrate its work to the Pacific, there is no reason why it cannot become more successful there.

Beyond the challenges of aid effectiveness in the Pacific, the Aid Program could, should it wish to engage, maximise the learning associated with its appraisal data by working with researchers and providing them with additional variables that could be compared with effectiveness. There would seem to be particular scope for work on project management practices—this work has been conducted by other donors (particularly the World Bank) and returned useful findings. Even if such data cannot be gathered retrospectively for the Australian Aid Program, they could be gathered moving forward. Data on aid delivery modalities (whether aid is given via NGOs, contractors, governments or the like) would also be a useful future area of engagement. We tried to match OECD modality data with Australian Aid Program appraisal data but were unable to do so owing to variation in project names and codes. Further data work may help in overcoming this obstacle. Independent of the data the Aid Program produces, there is much additional work to be done using aid effectiveness data. The country specialisation and experience of different donors is another variable that could be calculated and added to existing data sets in future work.

For now, we have shown that useful findings can emerge from the analysis of Australian aid appraisals. We have shown that Australia is not a unique donor but that it does face some particular challenges in its work in the Pacific. Overcoming these challenges will be central to creating an aid program that is as effective as possible.

### DATA AVAILABILITY STATEMENT

The data that support the findings of this study and the Supporting Information Appendices are openly available in figshare at http://doi.org/10.6084/m9.figshare.11678118, reference number 11678118.

## Supporting information

Data S1: Supporting Information
