# Supplementary material for: Australian aid projects: What works, where projects work and how Australia compares
Source: Asia Pac Policy Stud. 2020 May 11;7(2):171–86. doi: 10.1002/app5.300 (PMC7496248; doi:10.1002/app5.300)
Supplement: Supplementary file 1 — Data S1: Supporting Information [file APP5-7-171-s001.docx]

# Online appendices

## Appendix 1 – data by donor

Table A1 shows donor name, number of projects and the start date of the earliest and most recent project in the dataset. (Although some projects come from as long ago as 1948, these projects are excluded from any analysis involving recipient country characteristics as, for most variables, data on recipient countries does not span that far back.)

#### Table A1 – Donor and project details

|  | **Project performance** | | | | | **Dates** | |
| --- | --- | --- | --- | --- | --- | --- | --- |
|  | **n** | **Mean** | **SD** | **Min** | **Max** | **Oldest project** | **Newest project** |
| Australia | 456 | 4.29 | 0.74 | 2 | 6 | 1988 | 2016 |
| ADB | 1736 | 3.90 | 1.03 | 1.5 | 6 | 1968 | 2014 |
| DFID | 1917 | 4.62 | 0.97 | 1.2 | 6 | 1987 | 2011 |
| GFATM | 581 | 4.75 | 1.23 | 1.5 | 6 | 2003 | 2012 |
| GiZ | 129 | 4.46 | 0.87 | 2 | 6 | 1989 | 2009 |
| IFAD | 33 | 4.18 | 0.73 | 2 | 5 | 1993 | 2010 |
| JICA | 716 | 4.99 | 1.20 | 1.5 | 6 | 1981 | 2008 |
| KfW | 2021 | 4.16 | 1.11 | 1 | 6 | 1963 | 2010 |
| World Bank | 10119 | 4.05 | 1.23 | 1 | 6 | 1948 | 2016 |
| Total | 17708 | 4.18 | 1.19 | 1 | 6 | 1948 | 2016 |

Figure A1 – Effectiveness score distributions by donor


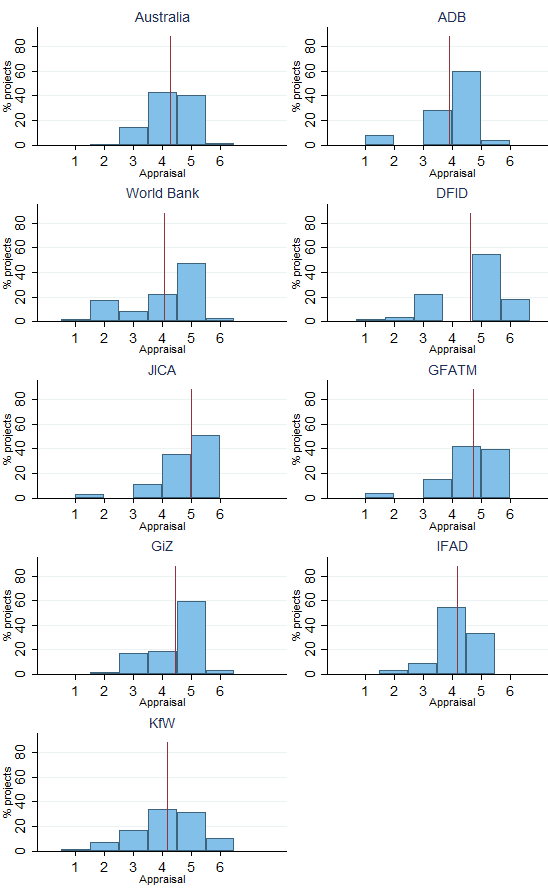


## Appendix 2 - the relationship between Australian aid effectiveness and other appraisal scores

AQC and FAQC reports assess projects on the following attributes:

- Effectiveness
- Efficiency
- Relevance
- Gender equality
- Monitoring and evaluation
- Sustainability

In the body of this paper we focused on effectiveness. To add to the analysis in the paper itself, the Table A2 shows correlation coefficients for the relationship between assessments of the different project attributes. The unit of analysis is the most recent AQC or FAQC for each project in the data provided to us. (Unlike in other analysis in this report, we also include regional projects. This is possible because there are no country-level variables. It is also desirable as it increases the sample size.)

#### Table A2 – Correlation between attributes

|  | **Effectiveness** | **Efficiency** | **Relevance** | **Gender** | **M & E** | **Sustainability** |
| --- | --- | --- | --- | --- | --- | --- |
| **Effectiveness** | 1.00 |  |  |  |  |  |
| **Efficiency** | 0.59*** | 1.00 |  |  |  |  |
| **Relevance** | 0.46*** | 0.39*** | 1.00 |  |  |  |
| **Gender Equality** | 0.42*** | 0.33*** | 0.26*** | 1.00 |  |  |
| **M & E** | 0.57*** | 0.53*** | 0.38*** | 0.50*** | 1.00 |  |
| **Sustainability** | 0.48*** | 0.44*** | 0.39*** | 0.35*** | 0.42*** | 1.00 |
| Observations | 585 |  |  |  |  |  |

Correlations can range between -1 and 1. A relationship of -1 would be a perfect negative correlation, 1 would be a perfect positive correlation. Zero indicates no relationship. In this comparison of attributes, all the attributes are positively correlated with each other. Typically, the relationship is quite strong. Projects that score well on one attribute tend to score well on the others. Projects that score less well on one attribute tend to score less-well on the others. All of the correlations are clearly statistically significant (p<0.01). None, are perfect, or even very close to perfect, but that is not surprising – perfect correlations rarely arise in social science. Additionally, the appraisal scores are ordinal rather than truly continuous, which likely reduces the nominal correlations somewhat.^[[1]](#footnote-1)^

Gender equality is the attribute least strongly correlated with the others. A clear relationship still exists. However, it would be useful in future work to study why the gender tends to be less strongly related than other attributes.

## Appendix 3 – appraisal of Australian project effectiveness in different countries

Figure A1 shows the mean appraisal of each Australian aid project in the Pacific broken down by recipient country. As noted in the main text of this report, Papua New Guinea scores surprisingly well, while Nauru scores poorly.

Figure A2 – Australian project effectiveness by Pacific country

Table A3 reports some key regression results with Papua New Guinea excluded. Most results are similar to those found in the equivalent PNG-inclusive table in the main body of this paper. The only obvious difference is that the distinction between humanitarian and non-humanitarian project performance covers a wider range of sectors with Papua New Guinea excluded.

Table A3 – Correlates of project outcomes with PNG excluded

|  | **Project** | **Project with FE** | **Country** | **Country with FE** | **Full** | **Full FE** |
| --- | --- | --- | --- | --- | --- | --- |
| Project size (natural log) | 0.08** | 0.08** |  |  | 0.07** | 0.06* |
|  | (0.03) | (0.03) |  |  | (0.03) | (0.04) |
| Duration of project (days) | -0.00 | -0.00 |  |  | -0.00 | -0.00 |
|  | (0.00) | (0.00) |  |  | (0.00) | (0.00) |
| Sector (humanitarian omitted) |  |  |  |  |  |  |
| Economic | -0.33*** | -0.31** |  |  | -0.33*** | -0.31** |
|  | (0.12) | (0.12) |  |  | (0.13) | (0.13) |
| Education | -0.22* | -0.23* |  |  | -0.21* | -0.22* |
|  | (0.12) | (0.12) |  |  | (0.12) | (0.12) |
| Governance | -0.27** | -0.28** |  |  | -0.26** | -0.27** |
|  | (0.12) | (0.12) |  |  | (0.13) | (0.13) |
| Health | -0.28* | -0.29** |  |  | -0.25* | -0.27* |
|  | (0.14) | (0.14) |  |  | (0.15) | (0.15) |
| Other | -0.34* | -0.41** |  |  | -0.33 | -0.40* |
|  | (0.20) | (0.21) |  |  | (0.21) | (0.21) |
| Civil & Political liberties |  |  | -0.02 | -0.02 | -0.01 | -0.01 |
|  |  |  | (0.01) | (0.01) | (0.02) | (0.02) |
| Government effectiveness |  |  | 0.01 | 0.07 | 0.01 | 0.07 |
|  |  |  | (0.11) | (0.11) | (0.11) | (0.11) |
| Real GDP per capita growth |  |  | -0.00 | -0.00 | -0.01 | -0.01 |
|  |  |  | (0.01) | (0.01) | (0.02) | (0.02) |
| Real GDP Per Capita (1000s) |  |  | 0.04** | 0.03** | 0.03** | 0.03* |
|  |  |  | (0.02) | (0.02) | (0.02) | (0.02) |
| Completion Year FE | No | Yes | No | Yes | No | Yes |
| Observations | 414 | 414 | 408 | 408 | 408 | 408 |
| Standard errors in parentheses |  |  |  |  |  |  |
| * p<0.10 ** p<0.05 *** p<0.01 | |  |  |  |  |  |

# Appendix 4 – Ordered logistic regressions

Table A4 replicates the results from Table 2 in the body of the paper. In this case, however, ordered logistic regressions are used instead of OLS. Ordered logistic regressions could not be computed with finish year fixed effects. However, results from other models are substantively the same as those in the main text.

#### Table A4 – ordered logistic regression results

|  | **Project** | **Country** | **Full** |
| --- | --- | --- | --- |
| Project size (natural log) | 0.20*** |  | 0.17** |
|  | (0.07) |  | (0.07) |
| Duration of project (days) | -0.00 |  | -0.00 |
|  | (0.00) |  | (0.00) |
| Sector (humanitarian omitted) |  |  |  |
| Economic | -0.65** |  | -0.66** |
|  | (0.31) |  | (0.32) |
| Education | -0.43 |  | -0.40 |
|  | (0.30) |  | (0.31) |
| Governance | -0.68** |  | -0.65** |
|  | (0.30) |  | (0.32) |
| Health | -0.58* |  | -0.51 |
|  | (0.35) |  | (0.35) |
| Other | -0.67 |  | -0.60 |
|  | (0.47) |  | (0.47) |
| Freedom |  | -0.04 | -0.03 |
|  |  | (0.04) | (0.04) |
| Government effectiveness |  | 0.01 | 0.02 |
|  |  | (0.26) | (0.26) |
| GDP growth |  | 0.00 | -0.01 |
|  |  | (0.03) | (0.04) |
| GDP Per Capita (1000s) |  | 0.09** | 0.08** |
|  |  | (0.04) | (0.04) |
| Observations | 456 | 450 | 450 |
| Robust standard errors in parentheses | | | |
| * p<0.10 ** p<0.05 *** p<0.01 | | | |

1. The correlation coefficients are Pearson correlation coefficients. Alternative methods such as polychoric correlations could have been used to produce this table and may have been more appropriate given the ordinal nature of the data. However, simple Pearson correlation coefficients are sufficient to demonstrate the nature of the relationship between the attribute appraisals. [↑](#footnote-ref-1)
